# Liquid Chromatography with Dual Mass Spectrometry Detection: An Approach to the Determination of Br-Containing Disinfection By-Products in Drinking Water

**DOI:** 10.3390/ijms27010386

**Published:** 2025-12-30

**Authors:** Sergey A. Sypalov, Ilya S. Varsegov, Eleonora V. Danilova, Nikolay V. Ulyanovskii, Dmitry S. Kosyakov, Margarita Yu. Vozhdaeva, Alfiya R. Kholova, Dmitrii M. Mazur, Albert T. Lebedev

**Affiliations:** 1 Laboratory of Environmental Analytical Chemistry, Core Facility Center “Arktika”, M.V. Lomonosov Northern (Arctic) Federal University, Northern Dvina Embankment 17, Arkhangelsk 163002, Russia; sypych.one@yandex.ru (S.A.S.); i.varsegov@narfu.ru (I.S.V.); danilovaelya2000@mail.ru (E.V.D.); n.ulyanovsky@narfu.ru (N.V.U.); d.kosyakov@narfu.ru (D.S.K.); mocehops@yandex.ru (A.T.L.); 2 State Unitary Enterprise “Ufavodokanal”, Water Treatment Station, Rossiyskaya St. 157/2, Ufa 450098, Russia; vozhdaeva@mail.ru (M.Y.V.); al-pochta@mail.ru (A.R.K.); 3 Faculty of Materials Science, Shenzhen MSU-BIT University, Shenzhen 517182, China; 4 Chemistry Department, Lomonosov Moscow State University, Leninskie Gory 1/3, Moscow 119991, Russia

**Keywords:** aqueous chlorination, brominated DBPs, HPLC-ICP-MS, HPLC-HRMS

## Abstract

Detecting and quantifying disinfection by-products (DBPs), especially brominated species (Br-DBPs), is analytically challenging, often necessitating multiple techniques and specific standards for each target. This complexity hinders comprehensive assessment. To overcome these limitations, we present a powerful, integrated approach combining liquid chromatography with inductively coupled plasma mass spectrometry (LC-ICP-MS) and high-resolution mass spectrometry (LC-HRMS). This method enables rapid, non-targeted group screening of Br-DBPs: LC-ICP-MS selectively identifies bromine-containing compounds, while LC-HRMS provides tentative structural identification. Crucially, this synergistic combination allows for the quantification of any Br-DBP without requiring individual reference standards. This study successfully demonstrates the application of this combined LC-ICP-MS and LC-HRMS strategy for the non-targeted detection, identification, and subsequent quantification of Br-DBPs in real drinking water samples, offering a significant advancement for DBP monitoring and risk assessment.

## 1. Introduction

Natural water from unpolluted natural sources is not safe for direct human consumption, as it may contain harmful microorganisms (bacteria, viruses, and parasites), as well as naturally occurring substances (certain minerals or organic matter) that may pose health risks if consumed untreated. Drinking untreated natural water can lead to various health issues, including gastrointestinal infections (causing diarrhea, vomiting, etc.), and in severe cases, more serious diseases. Diarrhea caused by unsafe water, sanitation, and hygiene results in an estimated 829,000 deaths annually worldwide [1]. Hence, modern water treatment plants include a multi-stage purification process. Coagulation, sedimentation, filtration, and adsorption is followed by the last and most important stage—disinfection, which removes dangerous and pathogenic microorganisms [2]. Among various approaches, applied [3,4] chlorination is the most widely used and common method of disinfection today, using chlorine-based reagents such as Cl_2_ and NaClO. Despite its low cost and high effectiveness, this method has a significant disadvantage—formation of disinfection by-products (DBPs). Starting from chloroform, which was the first discovered DBP [5], a whole range of chlorinated organic compounds was further discovered to appear in drinking water due to the chlorination process [6].

Chlorination by-products arise due to the interaction of active chlorine and dissolved organic matter of natural, so anthropogenic, origin. The main products of disinfection revealing the deep transformation of initial substrates mostly belong to volatile compounds such as trihalomethanes (THMs), haloacetonitriles (HANs), haloacetamides (HAcAms), haloacetic acids (HAAs), and others [7,8,9,10,11,12,13]. In this regard, only a narrow range of organic compounds is under control in developed countries [14]. These compounds typically include chlorinated derivatives; however, the presence of bromide, bromate ions, or bromine-containing organic compounds in water, as well as bromine-containing impurities in the disinfection reagent, can lead to the formation of bromine-containing disinfection by-products (Br-DBPs) [15,16,17,18]. These compounds often have an order of magnitude higher toxicity than their chlorine-containing analogues [19,20].

Despite numerous studies and many years of research in this field, new disinfection by-products are still being discovered every year. Modern estimations claim about 700–800 compounds have been identified as DBPs [21,22,23]. Discovery of new DBPs is commonly associated with the application of modern analytical techniques like gas chromatography or liquid chromatography combined with mass spectrometry (GC-MS and LC-MS) [11,14,23]. The trend in increasing the number of DBPs discovered is especially evident in studies where LC-MS is applied, as it may cover a larger variety of polar organic compounds passing through the column, while application of GC-MS limits the range of detectable compounds to low-molecular-weight, low-polar, and volatile/semivolatile substances. High-performance liquid chromatography combined with high-resolution mass spectrometry (HPLC-HRMS) is widely used for non-targeted screening and identification of new DBPs [24,25,26,27]. With all its benefits, it has low selectivity to bromine-containing disinfection by-products and requires a significant amount of labor, including sample preparation and data processing to detect and identify these new compounds. One of the ways to solve this problem involves the combination of HPLC-HRMS with inductively coupled plasma mass spectrometry (ICP-MS), which provides superior selectivity and sensitivity for Br. Hence, HPLC-HRMS together with the HPLC-ICP-MS technique could lead to more efficient and accurate detection of Br-DBPs, which would be beneficial for environmental monitoring and protection. Indeed, combination of these two methods has been demonstrated to enhance both the identification results as well as the selectivity and sensitivity to a large range of environmental organic pollutants, drugs, and their metabolites containing various elements (halogens, P, S, Se, As, etc.) [28,29,30,31,32,33,34,35,36]. Such an approach allowed not only the search for and identification of metabolites, but also the performance of quantitative analysis (without the need for reference samples or standards) using the isotopic dilution technique. Br-containing drugs and their metabolites in particular were thoroughly studied earlier [37,38,39,40,41]. However, application of combined LC-HRMS with the LC-ICP-MS technique for new Br-PBDs discovery and the study of transformation products is limited. Recently, we have demonstrated that Umifenovir, an antiviral medication containing bromine, which was widely used during the COVID-19 pandemic, produced a large variety of Br-PBDs [42,43]. The developed analytical method enabled identifying and quantifying the initial drug and three of its bromine-containing derivatives in wastewater and sewage sludge. Due to the lack of commercially available standards, the only option to carry out the quantitation of the transformation products was by the consistent analytical response of ICP-MS to bromine, regardless of the structure of the compounds analyzed.

Thus, the combination of LC-ICP-MS and LC-HRMS methods has great prospects and advantages for rapid group non-targeted screening of bromine-containing compounds, including Br-DBPs. The LC-ICP-MS method allows selective screening for bromine-containing compounds, while HPLC–HRMS provides tentative identification and assumption of the structure. Together, this opens up the possibility of quantitative analysis of any bromine-containing compound without individual reference samples.

This study demonstrates the application of a combination of LC-ICP-MS and LC-HRMS techniques for the detection, identification, and quantification of untargeted Br-DBPs in drinking water. The Ufa drinking water was chosen as the subject of this study, since it has been monitored by the State Unitary Enterprise “Ufavodokanal” for many years, during which a significant amount of data has been collected [44]. Furthermore, due to the presence of bromide ions naturally occurring in Ufa River water, low-molecular volatile Br-DBPs regularly appear in the drinking water.

## 2. Results and Discussion

The HPLC-ICP-MS method is highly selective for Br, which allows for a rapid, non-targeted search for bromine-containing compounds. Due to this advantage, it could help in the quick search for bromine precursors and bromine-containing disinfection by-products. On the other hand, structural information in such an approach is lost due to the complete decay of the organobromine precursors in the high-temperature plasma. To overcome this limitation, the HPLC-ESI-HRMS method with similar chromatographic parameters was used. The retention times of the analytes allowed speeding up the data processing and reliability of the identification process. Based on the accurate *m*/*z* values and isotope ratios of the ions, the formula of the non-target Br-DBPs was obtained, while collision-induced dissociation (CID) mass spectra revealed their possible structure.

### 2.1. Screening of Br-Containing Compounds

**Figure 1:**
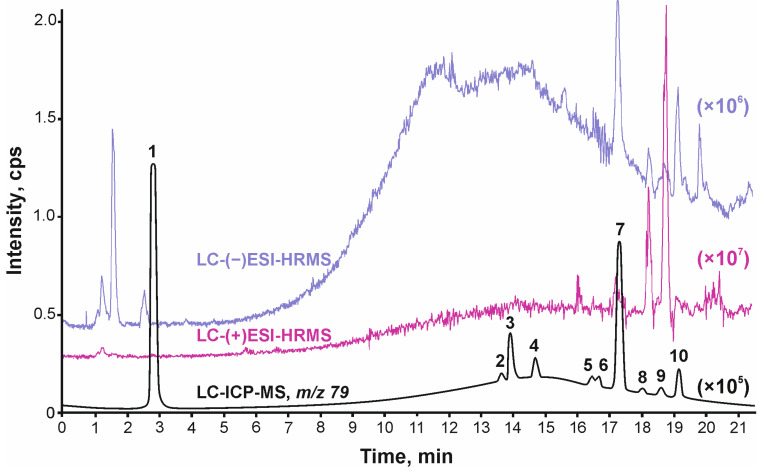
Chromatograms of PWI sample obtained by LC-ICP-MS (*m*/*z* 79, black line), LC-(+)ESI-HRMS (red line), and LC-(−)ESI-HRMS (blue line). 1–10 correspond to detected Br-containing compounds listed in Table 1.

Analysis of drinking water extracts by means of HPLC-ICP-MS has revealed the high efficiency of such an approach in the non-targeted search for bromine-containing compounds. Due to the enhanced sensitivity and selectivity of ICP-MS for bromine, at least 10 chromatographic peaks corresponding to Br-DBPs were easily detected in the chromatograms of water samples from the surface intake (PWI) and infiltration intake (IWI) (Figure 1). Similar chromatographic separation conditions allow performing a direct comparison of the same samples analyzed with (+/−)ESI-HRMS in information-dependent analysis (DDA) and ICP-MS. So, the peak finding procedure for Br-DBPs is demonstrated to be significantly easier in the ion chromatogram obtained for ICP-MS analysis. This feature helps further to focus the data processing procedure of ESI-HRMS results to particular retention time (RT) regions specific for Br-containing compounds (Figure 1), rather than processing the whole file by both manual or automatic algorithms.

**Figure 2:**
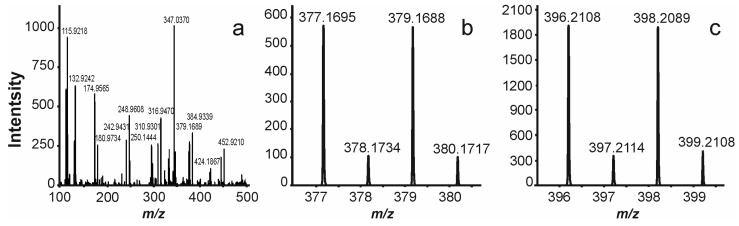
(**a**) (−)ESI mass spectrum covering RT 17.9–18.1 min of PWI sample, (**b**) cluster of [M − H]^−^ ion in the region of *m*/*z* 377–381, (**c**) cluster of [M + NH_4_]^+^ ion in the region of *m*/*z* 396–399.

For example, LC-ICP-MS mass chromatogram reveals a small peak (**8**) at RT 18.0 min belonging to some Br-containing compound. ESI-HRMS chromatograms, on the contrary, do not show any remarkable peak in this region. However, a targeted search at RT 17.9–18.1 min for any Br-containing signals in the ESI mass spectra (Figure 2a) demonstrates a characteristic isotopic pattern for bromine observed at *m*/*z* 377.1695 (C_18_H_34_BrO_3_, −0.5 ppm, [M − H]^−^ ion) in the negative ion mode (Figure 2b) and *m*/*z* 396.2108 (C_18_H_39_BrNO_3_, 0.04 ppm, [M + NH_4_]^+^ ion) in the positive ion mode (Figure 2c). Further, using the fragmentation pattern observed in the tandem mass spectrum (Figure S16) tentative structure could be proposed.

Thus, the advantages of combining these methods dramatically simplify the search and reliable detection of bromide-based disinfection products in drinking water. In this approach, LC-ICP-MS is used for a rapid, non-targeted search for bromine-containing compounds, while LC-ESI-HRMS is used for tentative identification of detected compounds.

### 2.2. Tentative Identification

Using the approach described above, elemental formulae (Tables S1–S9) and preliminary structures were determined for 10 Br-DBPs detected in water samples (Table 1).

The most abundant chromatographic peak eluting at the very beginning of the LC-ICP-MS chromatogram (Figure 1) contained a lot of signals in the corresponding (-)ESI mass spectrum with very rich isotopic patterns (Figure S1). Despite the fact of the evident presence of Br or Cl in these signals, most of them correspond to various bromide and chloride adducts formed with an iron ion (FeCl_3_^−^ *m*/*z* 160.8423, 3.4 ppm; FeCl_4_^−^ *m*/*z* 195.8113, 2.0 ppm; FeBr_3_Cl^−^ *m*/*z* 239.7612, 3.4 ppm; FeBr_2_Cl_2_^−^ *m*/*z* 283.7105, 2.2 ppm; etc.). Formation of such clusters is mostly due to the interaction of remaining bromide ions with iron from the instrument manifold, hence representing low interest in terms of the search for new Br-DBP.

Compounds corresponding to peaks **2**, **3**, **4**, and **5** were detected only in the negative ion mode. Based on their accurate masses and CID spectra (Figures S2–S10), they were identified as dibromonitrophenol (**2**), bromochloronitrophenol (**3**), tribromophenol (**4**), and dibromohydroxybenzoic acid (**5**). The formation and occurrence of these bromine-containing disinfection by-products has been previously demonstrated [18,45,46]. Though we were not able to fully describe the position of Br-atoms in the benzene ring of each isomer observed (Figure S8), we can suppose the particular isomer (for instance, 2,4,6-tribromophenol) in the case of the major product, according to general driving forces taking place during the aromatic electrophilic substitution reaction [47].

Compounds **6** and **7** were identified by ESI-HRMS in positive ion modes. Based on the data obtained, these compounds are likely to be hydroxybromooctadecenamide (**6**) and hydroxybromooctadecanamide (**7**), respectively. These products are formed through the conjugated electrophilic addition (Ad_E_) reaction of aqueous Br_2_ or HBrO with the double C=C bond of oleamide and linoleamide. Earlier, we have demonstrated this group of compounds to be a new class of DBPs present in the drinking water [48]. Hence, based on the mass spectra obtained before, we could assume their identity in the current analysis (Figures S11–S14). A compound of similar nature (hydroxybromooctadecanoic acid, *m*/*z* 377.1695, −0.5 ppm) was identified (Figures S15 and S16), as described above (peak **8**). The most probable pathway of this DBP formation also involves an Ad_E_ reaction with oleic acid.

**Figure 3:**
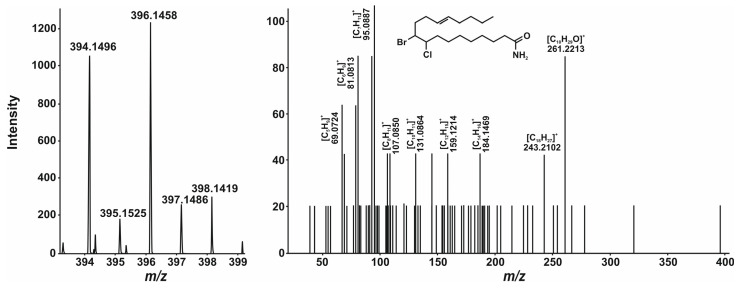
(+)ESI mass spectrum and its corresponding CID mass spectrum of compound **9**.

**Figure 4:**
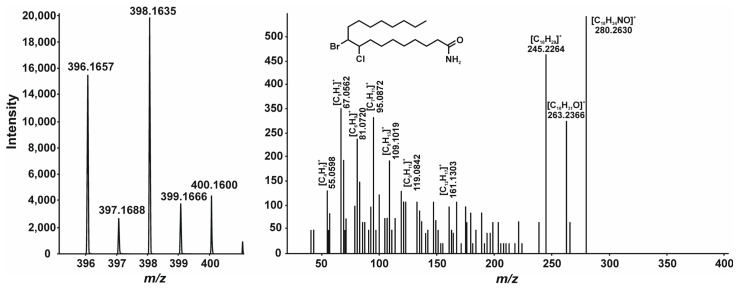
(+)ESI mass spectrum and CID mass spectrum of compound **10**.

In addition to the well-known disinfection by-products, new compounds (chlorobromooctadecenamide, **9**) and (chlorobromooctadecanamide, **10**) were found in the samples (Figures S17–S20). Although fragment ions observed in their CID spectra do not elucidate each halogen position, together with their accurate masses and isotopic distribution (Figure 3 and Figure 4), it was possible to propose a reasonable structure. Moreover, it may be justified by the earlier discovery of the formation of dichlorooctadecanamide and dichlorooctadecenamide during the water disinfection procedure [48]. These compounds are formed from oleamide and linoleamide by a similar conjugated addition reaction. However, the detection of mixed (Br-Cl) compounds **9** and **10** was not previously mentioned.

### 2.3. Quantitation Procedure

In addition to the non-targeted screening of Br-DBPs, the use of ICP-MS in combination with ESI-HRMS offers the opportunity for quantitative analysis of detected compounds using a single analytical standard. This approach was previously applied in the study of environmental samples for the transformation products of umifenovir during aqueous chlorination [42]. Standard solutions of umifenovir were analyzed over a wide range of concentrations, and the sensitivity coefficient of ICP-MS was calculated to determine the bromine dose dependence (based on the chromatographic peak area). It is worth mentioning that any bromine-containing compound may be used as standard. The lower detection limit for bromine was 0.2 ng/L [43], taking into account a concentrating factor of 2000. The number of replicates was 3 (n = 3; *p* = 0.95).

An equation was then developed to calculate the concentration of any bromine-containing compound that was detected and identified.

x = (y × M(C_l_H_m_Br_n_…))/(a_Br_ × M(Br_n_)),

y—the area of a chromatographic peak;
M(C_l_H_m_Br_n_…)—the molecular mass of the detected Br-containing compound;
a_Br_—the sensitivity coefficient of ICP-MS to bromine Br^79^ = 5.75 × 10^5^;
M(Br_n_)—the total mass of Br-atoms in the compound n × 79.904 a.m.u.

According to the proposed equation, the concentrations of detected Br-DBPs were calculated (Table 2).

The long-term studies demonstrate that the average values of total organic bromine for trihalomethanes in Ufa tap water from 2002 to 2019 were 1.7 µg/L for surface water and 0.87 µg/L for infiltration intake water [44]. These values are similar to the bromine concentration at peak I (1.3–1.2 ± 0.1 µg/L) corresponding to various bromide adducts detected by ICP-MS. Hence, we can suppose this peak refers to bromide ion transferred from the water during the sample preparation procedure.

The lowest amounts were observed for dibromonitrophenol (**2**, C_6_H_3_Br_2_NO_3_), tribromophenol (**4**, C_6_H_3_Br_3_O), and dibromohydroxybenzoic acid (**5**, C_7_H_4_Br_2_O_3_). Concentrations of these compounds in the samples of drinking water did not exceed 0.081 µg/L. On the contrary, the Br-DBPs of a similar nature, bromochloronitrophenol (**3**, C_6_H_3_BrClNO_3_), stands out with a concentration an order of magnitude higher, ranging from 0.26 ± 0.03 to 0.63 ± 0.06 µg/L. The concentrations of related compounds (bromochlorophenols, bromochlorohydroxybenzaldehydes, and bromochlorohydroxybenzoic acids), ranging from 0.001 to 0.061 µg/L, previously reported in tap water and source water samples from East China [49] were consistent with results obtained in the current research. This concordance suggests that formation of Br-DBPs is a prevalent issue across diverse water supplies and geographical regions, rather than being specific to the water source examined herein.

The highest concentrations among all detected Br-DBPs were found for compounds **6–10**, with hydroxybromooctadecanamide (**7**, C_18_H_36_BrNO_2_) having the maximum value among all of them (from 3.6 ± 0.4 to 4.1 ± 0.4 µg/L) at both stations of water intake. At the same time, the observed concentration of its unsaturated analogue hydroxybromooctadecenamide (**6**, C_18_H_34_BrNO_2_) was 20 times lower and reached only 0.18 ± 0.02 µg/L.

Discussing the difference in the amount of Br-DBPs between the water intake stations (PWI and IWI) sample leads to hydroxybromooctadecanoic acid (**8**, C_18_H_35_BrO_3_) and chlorobromooctadecanamide (**10**, C_18_H_35_BrClNO). The concentration of product **8** in water at IWI is 2.5 times higher (0.22 ± 0.02 µg/L), while the concentration of product **10** is 4 times higher (2.2 ± 0.2 µg/L). Together with that, the concentration of the unsaturated analogue of product **10**, chlorobromooctadecenamide (**9**, C_18_H_33_BrClNO), similarly to compounds **7** and **6**, was lower, ranging from 0.20 ± 0.02 to 0.28 ± 0.03 µg/L.

## 3. Materials and Methods

### 3.1. Chemicals and Reagents

Methanol (for gradient HPLC, Khimmed, Moscow, Russia), formic acid (ACS reagent, ≥96%, PanReac, Barcelona, Spain), and deionized water (type I, 18.2 MΩ·cm, Milli-Q, Molsheim, France) were used to prepare the mobile phase for HPLC, and grade solutions. Dichloromethane (PanReac, Barcelona, Spain), sulfuric acid, and sodium hydroxide (Komponent-Reaktiv, Moscow, Russia) were used for extraction.

### 3.2. Water Samples and Sample Preparation

During the spring floods of 2021, samples of incoming river water and prepared drinking water were collected at the surface and infiltration sampling stations of urban water treatment plants in Ufa. The RW sample represented river water that was supplied to the sewage treatment plant’s surface water intake, while the PWI sample was the prepared drinking water from the surface water intake that had undergone UV irradiation, primary chlorination, coagulation with aluminum sulfate, flocculation with polyacrylamide, sedimentation, rapid filtration through baked clay filters, and secondary chlorination. The IWI sample represented the prepared drinking water that had been obtained from infiltration wells and undergone single-stage disinfection using molecular chlorine.

The extraction of analytes from river and drinking water was performed using liquid–liquid extraction (LLE) based on the EPA 8270 method [50]. A sample of water (2 L) was adjusted to a pH of 2 by adding concentrated sulfuric acid and placed in a separating funnel together with 120 mL of dichloromethane. After shaking for 15–20 min, the organic solvent layer was separated and poured into a conical flask with a tightly fitting lid. The pH was then adjusted to 10 using a 10% sodium hydroxide solution, and the extraction process was repeated. The combined dichloromethane extract was then evaporated until dry, and the dry residue was dissolved in 1 mL of methanol. The solution was filtered through a nylon membrane filter with a pore size of 0.2 µm and placed into a vial for further HPLC-ICP-MS and HPLC-MS/MS analysis. The final concentration was 2000 times the original concentration.

### 3.3. LC-ICP-MS and LC-HRMS Analysis

Chromatographic separation was achieved on a Nucleodur PFP column (150 × 2 mm) with a pentafluorophenyl stationary phase (Macherey-Nagel, Düren, Germany) and a particle size of 1.8 µm, using an LC-30 Nexera HPLC system (Shimadzu, Kyoto, Japan). A mobile phase consisting of a 0.1% aqueous formic acid solution (A) and methanol (B) was used with the following gradient elution program: 0–1 min: 25% B, 1–20 min: linear change in B to 100%, 20–25 min: 100% B, with a flow rate of 0.25 mL/min, the oven temperature was set to 40 °C, the injection volume was 5 µL.

The detection and quantitative analysis of bromine-containing analytes was carried out on an Aurora Elite HPLC-ICP-MS system (Bruker Daltonics, Bremen, Germany). High-purity hydrogen (40 mL/min) was used as a reaction gas in a collision reaction interface. Detection was performed in the single ion-monitoring (SIM) mode (*m*/*z* 79); the scanning time was 500 ms with the following parameters: HF generator power—1.6 kW, sampling depth—5 mm. High-purity argon was used as a plasma-forming, auxiliary, additional, and nebulizing gas at the flow rates of 18.0, 1.65, 0.23, and 0.8 L/min, respectively. The system control, data acquisition, and data processing were carried out using Quantum v.3.1 and Compass CDS software v.3.0 (Bruker Daltonics, Bremen, Germany).

Formula elucidation and identification of the analytes detected was carried out using a Triple-TOF 5600+ HPLC-MS/MS system (AB Sciex, Concord, ON, Canada) with a quadrupole-time-of-flight mass analyzer. The electrospray ionization (ESI) source was operating in both positive ((+)ESI) and negative ((−)ESI) ion modes within a mass range of 100–1000 Da. Tandem mass spectra produced by collision-induced dissociation were recorded in a data (information)-dependent acquisition (IDA) mode, with collision energy varying from 20 to 60 eV. The signal intensity of the precursor ion was at least 100 counts/s, and the scanning range was set to 20–1000 Da for MS/MS analysis. Detection was performed with the following ion source parameters: voltage—5500 V, ionization source temperature—300 °C, pressure of 40 psi for spraying and drying gases, and a curtain gas pressure of 30 psi. The elemental composition was determined based on the exact masses and isotopic distribution of ions, using the following constraints: the maximum number of atoms of C—100, H—300, O—20, N—10, Br—10, and Cl—10; the mass accuracy error was set to <5 ppm (MS) and <10 ppm (MS/MS); signal-to-noise ratio ≥ 10. The system control, data acquisition, and data processing were performed using Analyst v.1.8, PeakView v.2.2, MasterView v.2.0, and FormulaFinder v.2.2 software packages (AB Sciex, Concord, ON, Canada).

## 4. Conclusions

The occurrence of Br-DBPs in water samples could be easily revealed using a combination of LC-ICP-MS and LC-HRMS methods. Targeting to bromine-containing compounds could be achieved due to the high sensitivity of ICP-MS, while the accurate mass measurement and fragmentation pattern obtained with HRMS result in the structure elucidation of unknown components. This approach allows performing a non-target analysis of Br-containing analytes in water processed with disinfecting agents. The obtainable data are unique since both identification and quantitation become possible without having any standards. Including such an approach in the screening protocol of water from disinfection water plants may significantly increase the data quality, helping to optimize the procedure.

## Figures and Tables

**Table 1:** List of Br-DBPs detected in water samples.

№	t_R_, min	*m*/*z*, [M − H]^−^	*m*/*z*, [M + H]^+^	Molecular Formula	Error, ppm	Preliminary Identity
**1**	2.9	+	-	-	-	-
**2**	13.5	293.8402	-	C_6_H_3_Br_2_NO_3_	−1.7	dibromonitrophenol
**3**	13.9	249.8913	-	C_6_H_3_BrClNO_3_	0.4	bromochloronitrophenol
**4**	14.6	326.7652	-	C_6_H_3_Br_3_O	−2.8	tribromophenol
**5**	16.4	292.8455	-	C_7_H_4_Br_2_O_3_	0.2	dibromohydroxybenzoic acid
**6**	16.6	-	376.1841	C_18_H_34_BrNO_2_	−1.2	hydroxybromooctadecenamide
**7**	17.3	-	378.2002	C_18_H_36_BrNO_2_	0.05	hydroxybromooctadecanamide
**8**	18.0	377.1695	-	C_18_H_35_BrO_3_	−0.5	Hydroxybromooctadecanoic acid
**9**	18.6	-	394.1496	C_18_H_33_BrClNO	−2.7	chlorobromooctadecenamide
**10**	19.1	-	396.1657	C_18_H_35_BrClNO	−1.6	chlorobromooctadecanamide

**Table 2:** Concentration of Br-DBPs in samples of natural and drinking water.

№	Formula	Molecular Mass, a.m.u.	Mass Fraction of Br, %	Concentration in the Sample, µg/L		
				RW	PWI	IWI
**1**	Adducts with bromide ion	-	-	n.d.	1.3 ± 0.1 *	1.2 ± 0.1 *
**2**	C_6_H_3_Br_2_NO_3_	296.9028	54	n.d.	0.075 ± 0.007	0.033 ± 0.003
**3**	C_6_H_3_BrClNO_3_	252.4515	32	n.d.	0.63 ± 0.06	0.26 ± 0.03
**4**	C_6_H_3_Br_3_O	330.8012	72	n.d.	0.081 ± 0.008	0.035 ± 0.004
**5**	C_7_H_4_Br_2_O_3_	295.9150	54	n.d.	0.060 ± 0.006	0.058 ± 0.006
**6**	C_18_H_34_BrNO_2_	376.3775	21	n.d.	0.18 ± 0.02	0.18 ± 0.02
**7**	C_18_H_36_BrNO_2_	378.3934	21	n.d.	3.6 ± 0.4	4.1 ± 0.4
**8**	C_18_H_35_BrO_3_	379.3781	21	n.d.	0.087 ± 0.009	0.22 ± 0.02
**9**	C_18_H_33_BrClNO	394.8229	20	n.d.	0.20 ± 0.02	0.28 ± 0.03
**10**	C_18_H_35_BrClNO	396.8387	20	n.d.	0.55 ± 0.05	2.2 ± 0.2

*—total concentration of Br, n.d.—not detected.

## Data Availability

The original contributions presented in this study are included in the article/Supplementary Material. Further inquiries can be directed to the corresponding author.

## Supplementary Materials

The following supporting information can be downloaded at: https://www.mdpi.com/article/10.3390/ijms27010386/s1.

## References

[1] World Health Organization (2022). Water and Sanitation Fact Sheet. https://www.who.int/europe/news-room/fact-sheets/item/water-and-sanitation.

[2] Cutler D., Miller G. (2005). The role of public health improvements in health advances: The twentieth-century United States. Demography.

[3] (1999). Alternative Disinfectants and Oxidants Guidance Manual. https://www.epa.gov/dwreginfo/interim-enhanced-surface-water-treatment-rule-documents.

[4] Lebedev A.T. (2007). Mass spectrometry in the study of mechanisms of aquatic chlorination of organic substrates. Eur. J. Mass Spectrom..

[5] Rook J.J. (1974). Formation of haloforms during chlorination of natural waters. Water Treat. Exam..

[6] Bellar T.A., Lichtenberg J.J., Kroner R.C. (1974). Occurance of organohalides in chlorinated drinking waters. J. Am. Water Work. Assoc..

[7] Richardson S.D., Plewa M.J., Wagner E.D., Schoeny R., De-Marini D.M. (2007). Occurrence, genotoxicity, and carcinogenicity of regulated and emerging disinfection by-products in drinking water: A review and roadmap for research. Mutat. Res. Rev. Mutat. Res..

[8] Clayton G.E., Thorn R.M., Reynolds D.M. (2019). Comparison of Trihalomethane Formation Using Chlorine-Based Disinfectants Within a Model System; Applications Within Point-of-Use Drinking Water Treatment. Front. Environ. Sci..

[9] Roccaro P., Vagliasindi F.G., Korshin G.V. (2014). Relationships between trihalomethanes, haloacetic acids, and haloacetonitriles formed by the chlorination of raw, treated, and fractionated surface waters. J. Water Supply Res. Technol..

[10] Lei X., Xie Z., Sun Y., Qiu J., Yang X. (2023). Recent progress in identification of water disinfection byproducts and opportunities for future research. Environ. Pollut..

[11] Dong F., Zhu J., Li J., Fu C., He G., Lin Q., Li C., Song S. (2023). The occurrence, formation and transformation of disinfection byproducts in the water distribution system: A review. Sci. Total. Environ..

[12] Forster A.L., Wiskur S.L., Richardson S.D. (2025). Formation of Eight Classes of DBPs from Chlorine, Chloramine, and Ozone: Mechanisms and Formation Pathways. Environ. Sci. Technol..

[13] Mazur D.M., Lebedev A.T. (2022). Transformation of Organic Compounds during Water Chlorination/Bromination: Formation Pathways for Disinfection By-Products (A Review). J. Anal. Chem..

[14] Richardson S.D. (2021). Tackling unknown disinfection by-products: Lessons learned. J. Hazard. Mater. Lett..

[15] Richardson S.D., Thruston A.D., Rav-Acha C., Groisman L., Popilevsky I., Juraev O., Glezer V., McKague A.B., Plewa M.J., Wagner E.D. (2003). Tribromopyrrole, Brominated Acids, and Other Disinfection Byproducts Produced by Disinfection of Drinking Water Rich in Bromide. Environ. Sci. Technol..

[16] Powers L.C., Conway A., Mitchelmore C.L., Fleischacker S.J., Harir M., Westerman D.C., Croué L.C., Schmitt-Kopplin P., Richardson S.D., Gonsior M. (2020). Tracking the formation of new brominated disinfection by-products during the seawater desalination process. Environ. Sci. Water Res. Technol..

[17] Krasner S.W., Lee C.F.T., Chinn R., Hartono S., Weinberg H., Richardson S.D., Pressman J.G., Speth T.F., Miltner R.J., Simmons J.E. Bro-mine incorporation in regulated and emerging DBPs and the relative predominance of mono-, di-, and trihalogenated DBPs. Proceedings of the 2008 AWWA Water Quality Technology Conference.

[18] Pan Y., Zhang X. (2013). Four groups of new aromatic halogenated disinfection byproducts: Effect of bromide concentration on their formation and speciation in chlorinated drinking water. Environ. Sci. Technol..

[19] Plewa M.J., Wagner E.D., Muellner M.G., Hsu K.-M., Richardson S.D. (2008). Comparative mammalian cell toxicity of NDBPs and C-DBPs. Disinfection by-products in drinking water. ACS Symp. Ser..

[20] Yang M., Zhang X. (2013). Comparative developmental toxicity of new aromatic halogenated DBPs in a chlorinated saline sewage effluent to the marine polychaete Platynereis dumerilii. Environ. Sci. Technol..

[21] Richardson S.D., Postigo C. (2016). A new technique helps to uncover unknown peptides and disinfection by-products in water. J. Environ. Sci..

[22] Richardson S.D., Ternes T.A. (2018). Water analysis: Emerging contaminants and current issues. Anal. Chem..

[23] Yang M., Zhang X. (2016). Current trends in the analysis and identification of emerging disinfection byproducts. Trends Environ. Anal. Chem..

[24] Wu J., Zhang Y., Zhang Q., Tan F., Liu Q., Yang X. (2024). The Selectively Nontargeted Analysis of Halogenated Disinfection Byproducts in Tap Water by Micro-LC QTOFMS. Toxics.

[25] Richardson S.D., Kimura S.Y. (2020). Water Analysis: Emerging Contaminants and Current Issues. Anal. Chem..

[26] Richardson S.D., Ternes T.A. (2021). Water analysis: Emerging contaminants and current issues. Anal. Chem..

[27] Richardson S.D., Manasfi T. (2024). Water Analysis: Emerging Contaminants and Current Issues. Anal. Chem..

[28] Feldmann J., Raab A., Krupp E.M. (2018). Importance of ICPMS for speciation analysis is changing: Future trends for targeted and non-targeted element speciation analysis. Anal. Bioanal. Chem..

[29] Meermann B., Bockx M., Laenen A., Van Looveren C., Cuyckens F., Vanhaecke F. (2012). Speciation analysis of bromine-containing drug metabolites in feces samples from a human in vivo study by means of HPLC/ICP-MS combined with on-line isotope dilution. Anal. Bioanal. Chem..

[30] Hogeback J., Schwarzer M., Wehe C.A., Sperling M., Karst U. (2015). Investigating the adduct formation of organic mercury species with carbonic anhydrase and hemoglobin from human red blood cell hemolysate by means of LC/ESI-TOF-MS and LC/ICP-MS. Metallomics.

[31] Delafiori J., Ring G., Furey A. (2016). Clinical applications of HPLC–ICP-MS element speciation: A review. Talanta.

[32] Kokarnig S., Tsirigotaki A., Wiesenhofer T., Lackner V., Francesconi K.A., Pergantis S.A., Kuehnelt D. (2015). Concurrent quantitative HPLC–mass spectrometry profiling of small selenium species in human serum and urine after ingestion of selenium supplements. J. Trace Elem. Med. Biol..

[33] Jensen B.P., Gammelgaard B., Hansen S.H., Andersen J.V. (2005). HPLC-ICP-MS compared with radio-chemical detection for metabolite profiling of 3H-bromohexine in rat urine and faeces. J. Anal. At. Spectrom..

[34] Gammelgaard B., Hansen H.R., Stürup S., Møller C. (2008). The use of inductively coupled plasma mass spectrometry as a detector in drug metabolism studies. Expert Opin. Drug Metab. Toxicol..

[35] Amayo K.O., Petursdottir A., Newcombe C., Gunnlaugsdottir H., Raab A., Krupp E.M., Feldmann J. (2011). Identification and Quantification of Arsenolipids Using Reversed-Phase HPLC Coupled Simultaneously to High-Resolution ICPMS and High-Resolution Electrospray MS without Species-Specific Standards. Anal. Chem..

[36] Lorenc W., Kruszka D., Kachlicki P., Kozłowska J., Barałkiewicz D. (2020). Arsenic species and their transformation pathways in marine plants. Usefulness of advanced hyphenated techniques HPLC/ICP-MS and UPLC/ESI-MS/MS in arsenic species analysis. Talanta.

[37] Jensen B.P., Smith C.J., Bailey C.J., Rodgers C., Wilson I.D., Nicholson J.K. (2005). Investigation of the metabolic fate of 2-, 3- and 4-bromobenzoic acids in bile-duct-cannulated rats by inductively coupled plasma mass spectrometry and high-performance liquid chromatography/inductively coupled plasma mass spectrometry/electrospray mass spectrometry. Rapid Communications in Mass Spectrometry: An International Journal Devoted to the Rapid Dissemination of Up-to-the-Minute. J. Mass Spectrom..

[38] Abou-Shakra F.R., Sage A.B., Castro-Perez J., Nicholson J.K., Lindon J.C., Scarfe G.B., Wilson I.D. (2002). High-performance liquid chromatography-UV diode array, inductively coupled plasma mass spectrometry (ICMPS) and orthogonal acceleration time-of-flight mass spectrometry (oa-TOFMS) applied to the simultaneous detection and identification of metabolites of 4-bromoaniline in rat urine. Chromatographia.

[39] Cuyckens F., Balcaen L.I., De Wolf K., De Samber B., Van Looveren C., Hurkmans R., Vanhaecke F. (2008). Use of the bromine isotope ratio in HPLC-ICP-MS and HPLC-ESI-MS analysis of a new drug in development. Anal. Bioanal. Chem..

[40] Nicholson J.K., Lindon J.C., Scarfe G., Wilson I.D., Abou-Shakra F., Castro-Perez J., Eaton A., Preece S. (2000). High-performance liquid chromatography and inductively coupled plasma mass spectrometry (HPLC-ICP-MS) for the analysis of xenobiotic metabolites in rat urine: Application to the metabolites of 4-bromoaniline. Analyst.

[41] Nicholson J.K., Lindon J.C., Scarfe G.B., Wilson I.D., Abou-Shakra F., Sage A.B., Castro-Perez J. (2001). High-performance liquid chromatography linked to inductively coupled plasma mass spectrometry and orthogonal acceleration time-of-flight mass spectrometry for the simultaneous detection and identification of metabolites of 2-bromo-4-trifluoromethyl-[13C]-acetanilide in rat urine. Anal. Chem..

[42] Ul’yanovskii N.V., Kosyakov D.S., Sypalov S.A., Varsegov I.S., Shavrina I.S., Lebedev A.T. (2022). Antiviral drug Umifenovir (Arbidol) in municipal wastewater during the COVID-19 pandemic: Estimated levels and transformation. Sci. Total Environ..

[43] Sypalov S.A., Ul’yanovskii N.V., Kosyakov D.S., Lebedev A.T. (2023). Determination of Umifenovir and Its Metabolites by High-Performance Liquid Chromatography with Combined Mass Spectrometric Detection. J. Anal. Chem..

[44] Vozhdaeva M.Y., Kholova A.R., Melnitskiy I.A., Beloliptsev I.I., Vozhdaeva Y.S., Kantor E.A., Lebedev A.T. (2021). Monitoring and Statistical Analysis of Formation of Organochlorine and Organobromine Compounds in Drinking Water of Different Water Intakes. Molecules.

[45] Ding G., Zhang X., Yang M., Pan Y. (2013). Formation of new brominated disinfection byproducts during chlorination of saline sewage effluents. Water Res..

[46] Zhai H., Zhang X. (2011). Formation and decomposition of new and unknown polar brominated disinfection byproducts during chlorination. Environ. Sci. Technol..

[47] Detenchuk E.A., Mazur D.M., Latkin T.B., Lebedev A.T. (2022). Halogen substitution reactions of halobenzenes during water disinfection. Chemosphere.

[48] Kosyakov D.S., Ul’yanovskii N.V., Popov M.S., Latkin T.B., Lebedev A.T. (2017). Halogenated fatty amides–A brand new class of disinfection by-products. Water Res..

[49] Pan Y., Wang Y., Li A., Xu B., Xian Q., Shuang C., Shi P., Zhou Q. (2017). Detection, formation and occurrence of 13 new polar phenolic chlorinated and brominated disinfection byproducts in drinking water. Water Res..

[50] (2014). Semivolatile Organic Compounds by Gas Chromatography/Mass Spectrometry (GC/MS).

