# Supplementary material for: Liquid Chromatography with Dual Mass Spectrometry Detection: An Approach to the Determination of Br-Containing Disinfection By-Products in Drinking Water
Source: Int J Mol Sci. 2025 Dec 30;27(1):386. doi: 10.3390/ijms27010386 (PMC12785680; doi:10.3390/ijms27010386)
Supplement: Supplementary file 1 [file ijms-27-00386-s001.zip › ijms-4033544-supplementary.pdf]

Supplementary material

HPLC-ICP-MS and HPLC-HRMS Combination: A New Approach to the Screening and Determination of Br-Containing Disinfection By-Products in Drinking Water

Sergey A. Sypalov[1], Ilya S. Varsegov[1], Eleonora V. Danilova[1], Nikolay V. Ulyanovskii[1], Dmitry S. Kosyakov[1], Margarita Yu. Vozhdaeva[2], Alfiya R. Kholova[2], Dmitrii M. Mazur[3,4]*, Albert T. Lebedev[1]

[1] Laboratory of Environmental Analytical Chemistry, Core Facility Center "Arktika", M.V. Lomonosov Northern (Arctic) Federal University, Northern Dvina Emb. 17, 163002 Arkhangelsk, Russia; s.sipalov@narfu.ru (S.A.S.); i.varsegov@narfu.ru (I.S.V.), e.v.danilova@narfu.ru (E.V.D.); n.ulyanovsky@narfu.ru (N.V.U.); d.kosyakov@narfu.ru (D.S.K.); mocehops@yandex.ru (A.T.L.)
[2] State Unitary Enterprise "Ufavodokanal", Water Treatment Station, Rossiyskaya St. 157/2, Ufa 450098, Russia; al-pochta@mail.ru (A.R.K.); vozhdaeva@mail.ru (M.Y.V.)
[3] Department of Materials Science, Shenzhen MSU-BIT University, Shenzhen 517182, China
[4] Lomonosov Moscow State University, Chemistry Department, Leninskie Gory 1/3, Moscow, 119991, Russia

* Correspondence: MazurDM@my.msu.ru (M.D.M.)

Contents:

Figure S1. Mass spectrum of the Peak 1
Figure S2. Mass spectrum of the Product 2
Figure S3. Tandem mass spectrum of the Product 2
Table S1. Found elemental composition of the Product 2
Figure S4. Mass spectrum of the Product 3
Figure S5. Tandem mass spectrum of the Product 3
Table S2. Found elemental composition of the Product 3
Figure S6. Mass spectrum of the Product 4
Figure S7. Tandem mass spectrum of the Product 4
Figure S8. XIC chromatograms of the Product 4
Table S3. Found elemental composition of the Product 4
Figure S9. Mass spectrum of the Product 5
Figure S10. Tandem mass spectrum of the Product 5
Table S4. Found elemental composition of the Product 5
Figure S11. Mass spectrum of the Product 6
Figure S12. Tandem mass spectrum of the Product 6
Table S5. Found elemental composition of the Product 6
Figure S13. Mass spectrum of the Product 7
Figure S14. Tandem mass spectrum of the Product 7
Table S6. Found elemental composition of the Product 7
Figure S15. Mass spectrum of the Product 8
Figure S16. Tandem mass spectrum of the Product 8
Table S7. Found elemental composition of the Product 8
Figure S17. Mass spectrum of the Product 9
Figure S18. Tandem mass spectrum of the Product 9

Table S8. Found elemental composition of the Product 9
Figure S19. Mass spectrum of the Product 10
Figure S20. Tandem mass spectrum of the Product 10
Table S9. Found elemental composition of the Product 10

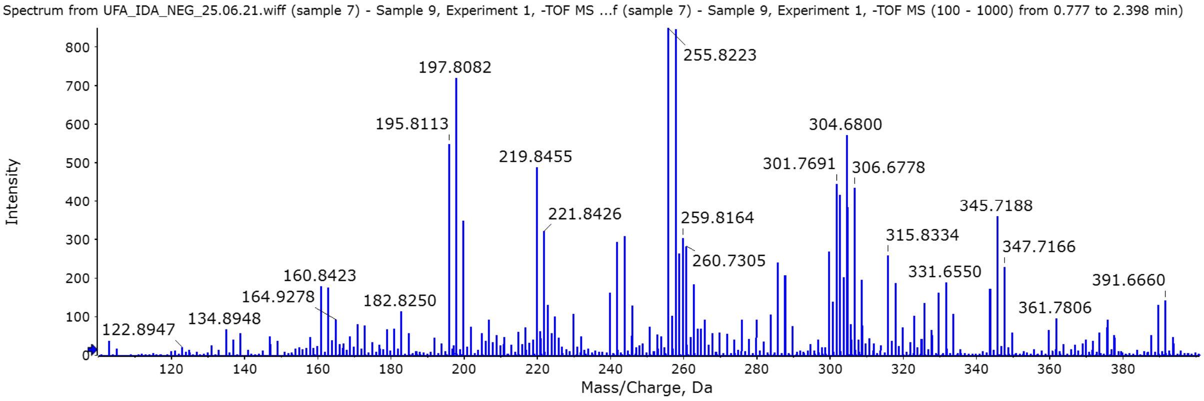

Figure S1. Mass spectrum of the Peak I

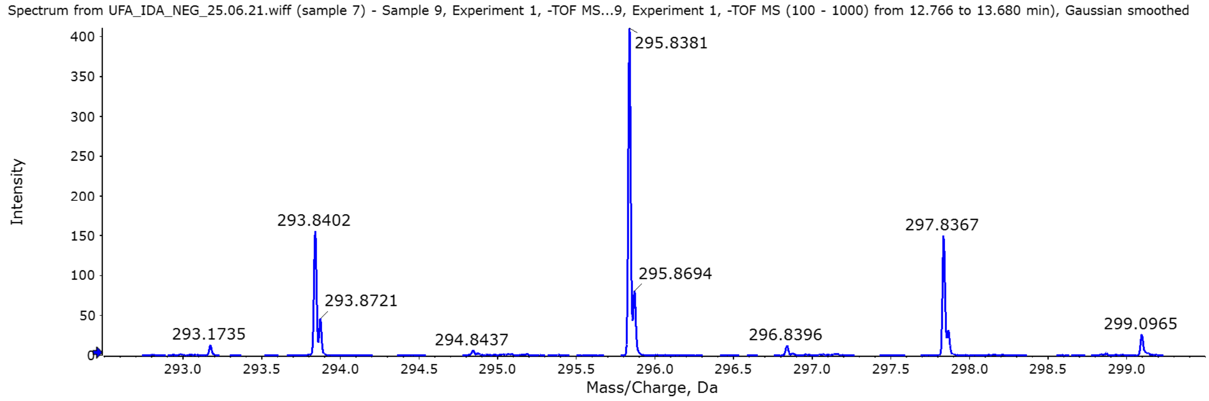

Figure S2. Mass spectrum of the Product II

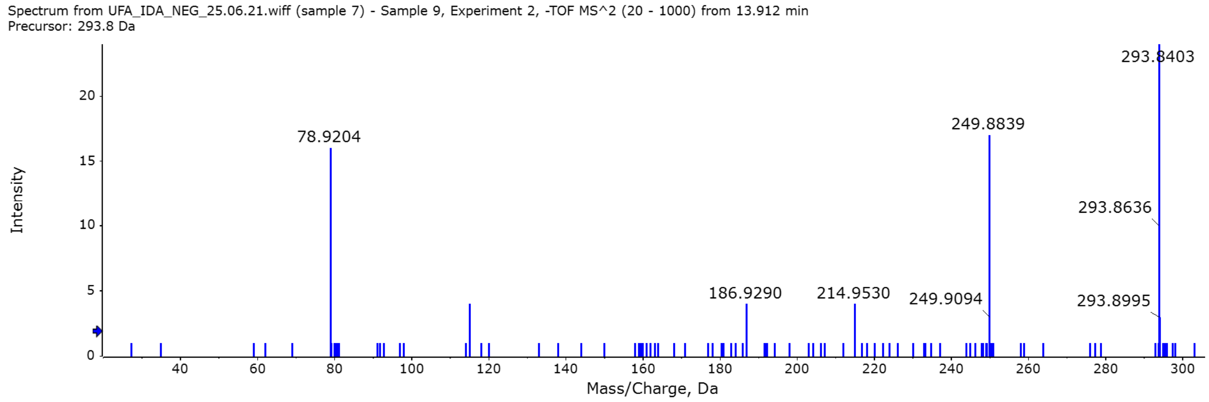

Figure S3. Tandem mass spectrum of the Product II

Table S1. Found elemental composition of the Product II

Hit	Formula	m/z	RDB	ppm	MS Rank	MSMS ppm	MSMS Rank	Found
1	$C_6H_3Br_2NO_3$	293.8407	5.0	-1.7	1	1.3 (1)	1	NA/NA

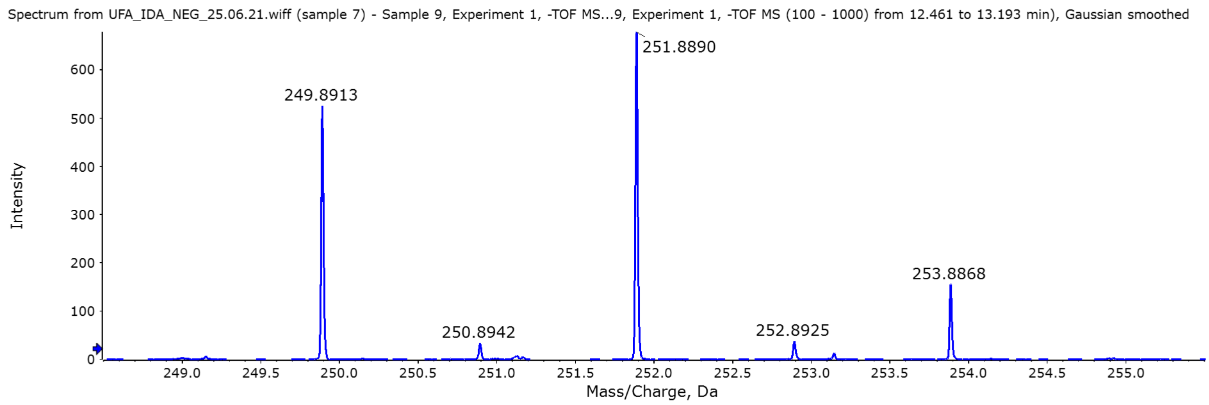

Figure S4. Mass spectrum of the Product 3

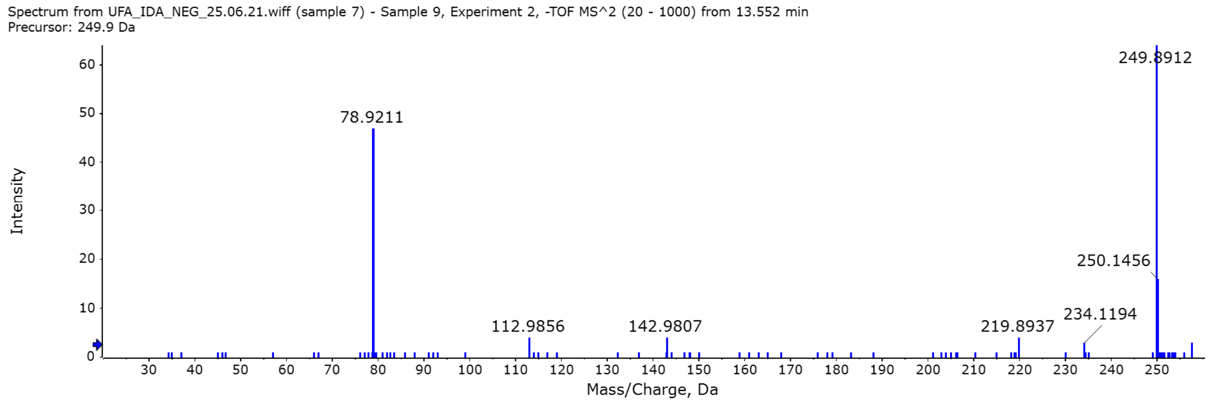

Figure S5. Tandem mass spectrum of the Product 3

Table S2. Found elemental composition of the Product 3

Hit	Formula	m/z	RDB	ppm	MS Rank	MSMS ppm	MSMS Rank	Found
1	$C_6H_3BrClNO_3$	249.8912	5.0	0.4	1	0.3 (2)	1	NA/NA

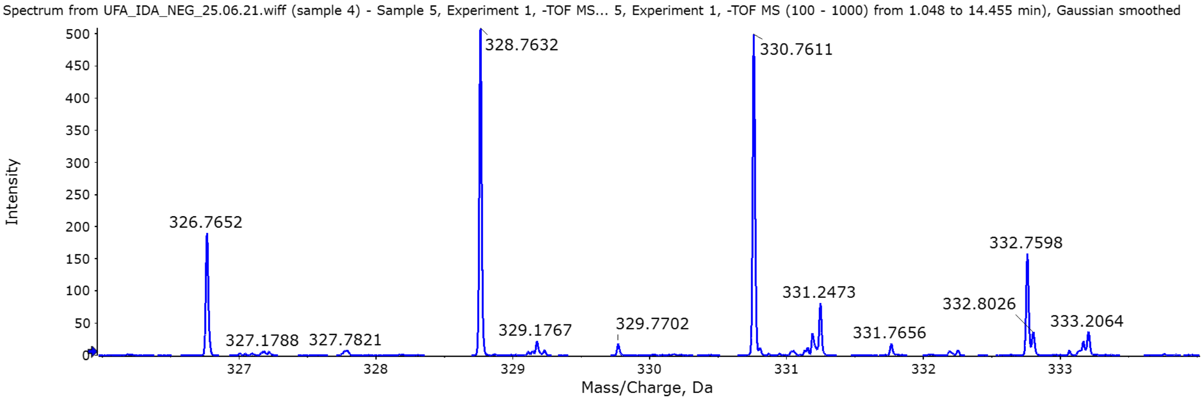

Figure S6. Mass spectrum of the Product 4

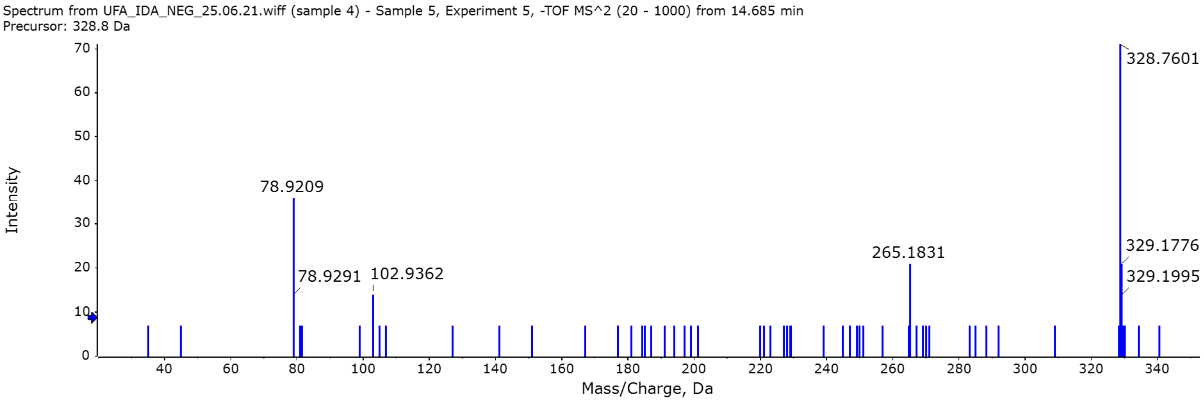

Figure S7. Tandem mass spectrum of the Product 4

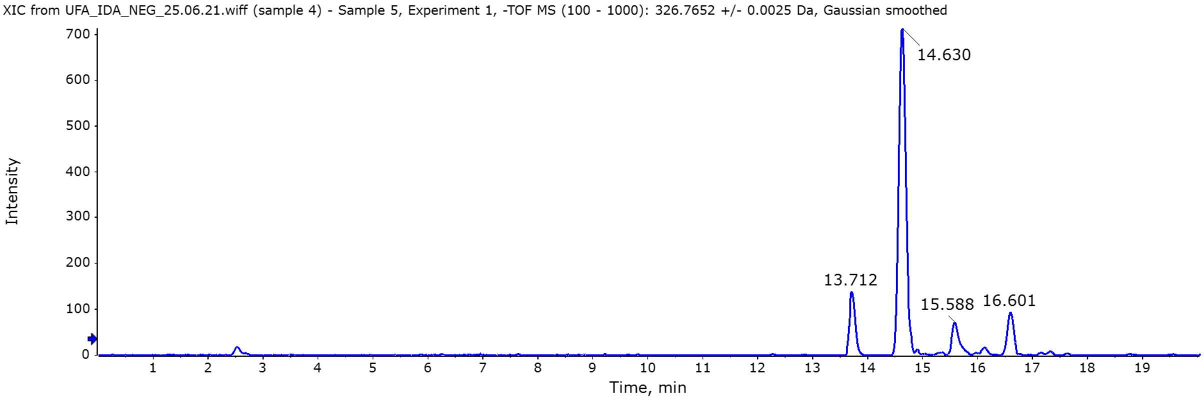

Figure S8. XIC chromatograms of the Product 4

Table S3. Found elemental composition of the Product 4

Hit	Formula	m/z	RDB	ppm	MS Rank	MSMS ppm	MSMS Rank	Found
1	$C_6H_3Br_3O$	326.7661	4.0	-2.8	1	-	1	NA/NA

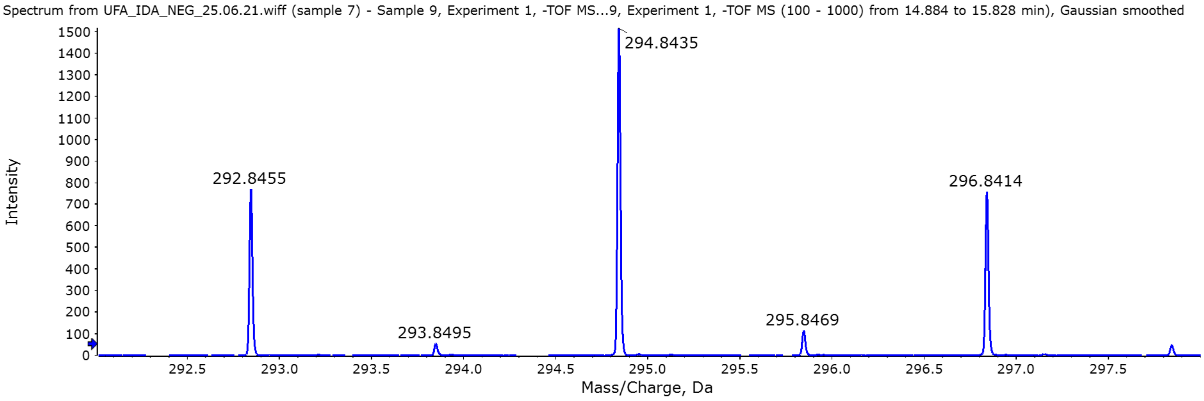

Figure S9. Mass spectrum of the Product 5

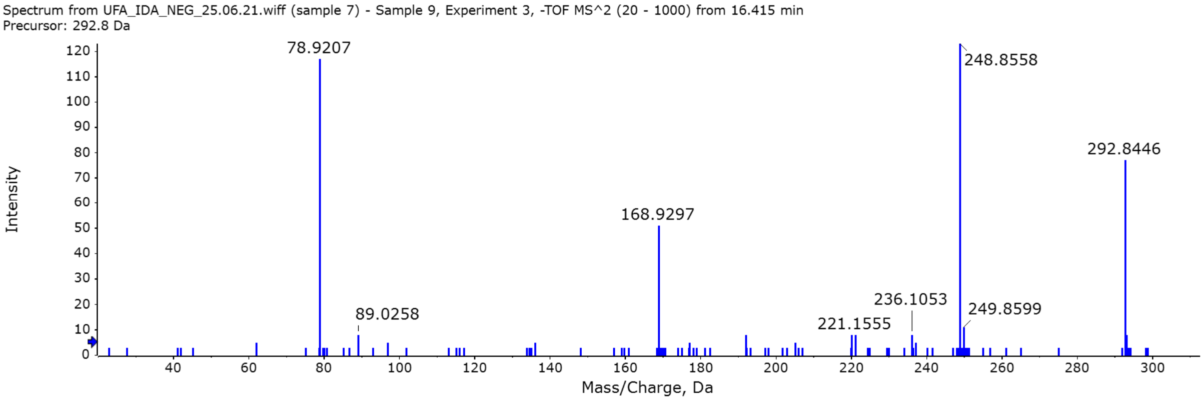

Figure S10. Tandem mass spectrum of the Product 5

Table S4. Found elemental composition of the Product 5

Hit	Formula	m/z	RDB	ppm	MS Rank	MSMS ppm	MSMS Rank	Found
1	$C_7H_4Br_2O_3$	292.8454	5.0	0.2	1	1.6 (3)	1	NA/NA

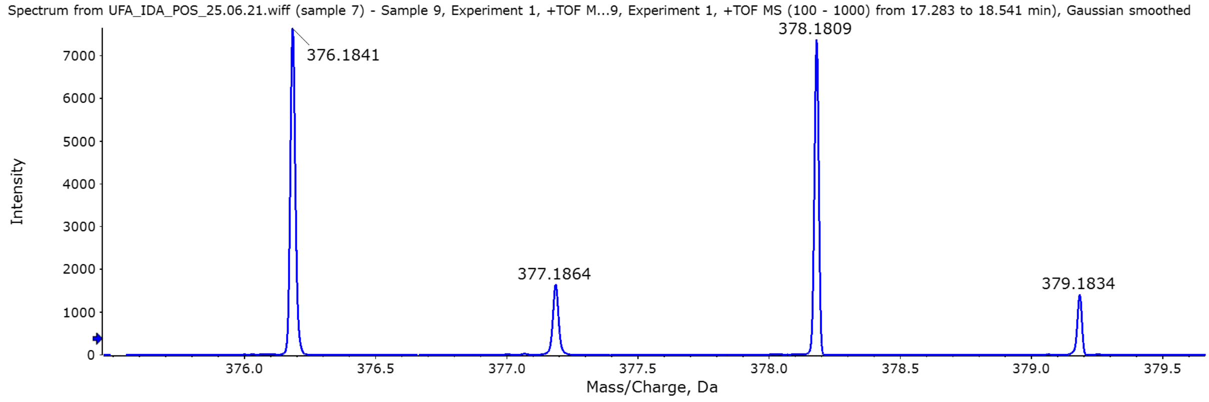

Figure S11. Mass spectrum of the Product 6

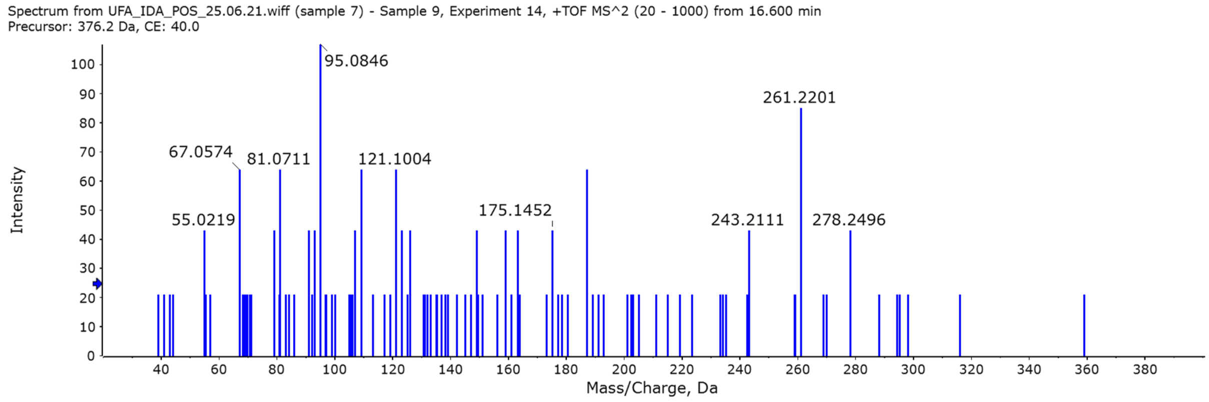

Figure S12. Tandem mass spectrum of the Product 6

Table S5. Found elemental composition of the Product 6

Hit	Formula	m/z	RDB	ppm	MS Rank	MSMS ppm	MSMS Rank	Found
1	$C_{18}H_{34}BrNO_2$	376.1846	2.0	-1.2	1	4.4 (2)	1	NA/NA

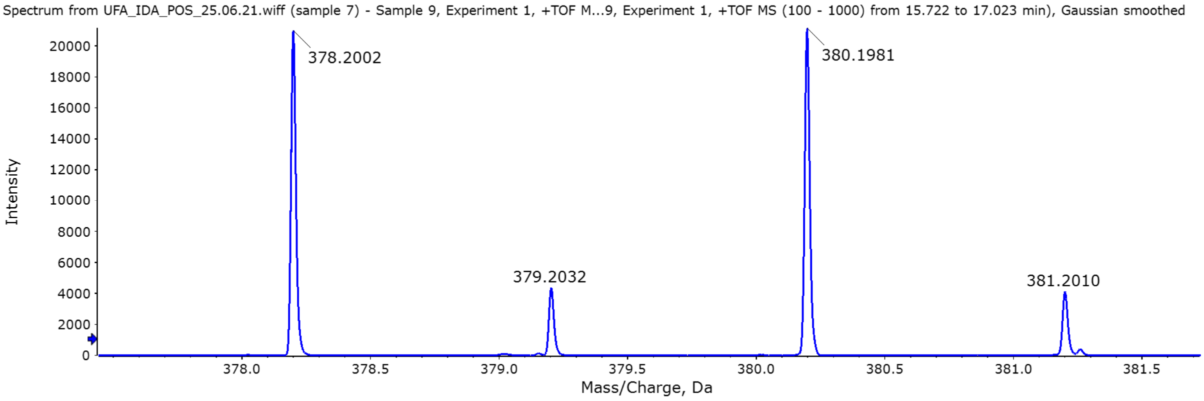

Figure S13. Mass spectrum of the Product 7

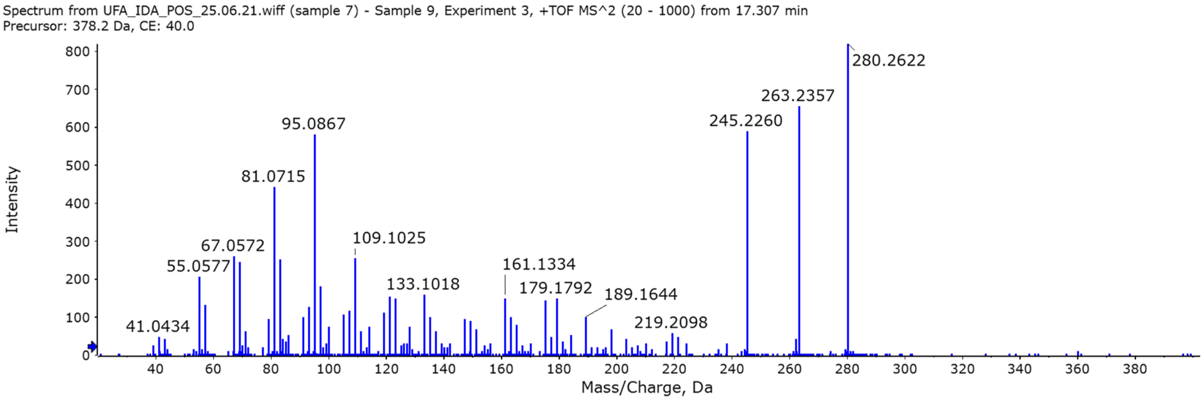

Figure S14. Tandem mass spectrum of the Product 7

Table S6. Found elemental composition of the Product 7

Hit	Formula	m/z	RDB	ppm	MS Rank	MSMS ppm	MSMS Rank	Found
1	$C_{18}H_{36}BrNO_2$	378.2002	1.0	0.05	1	3.7 (33)	1	NA/NA

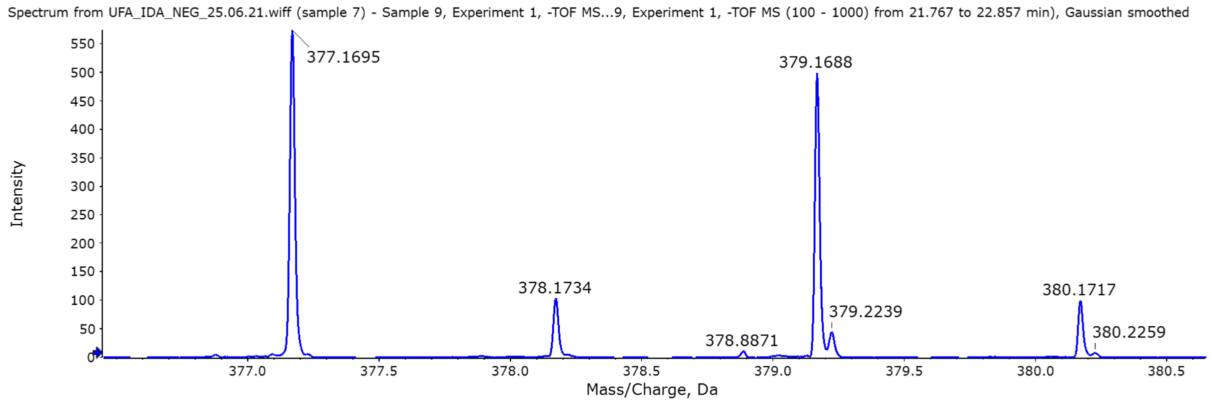

Figure S15. Mass spectrum of the Product 8

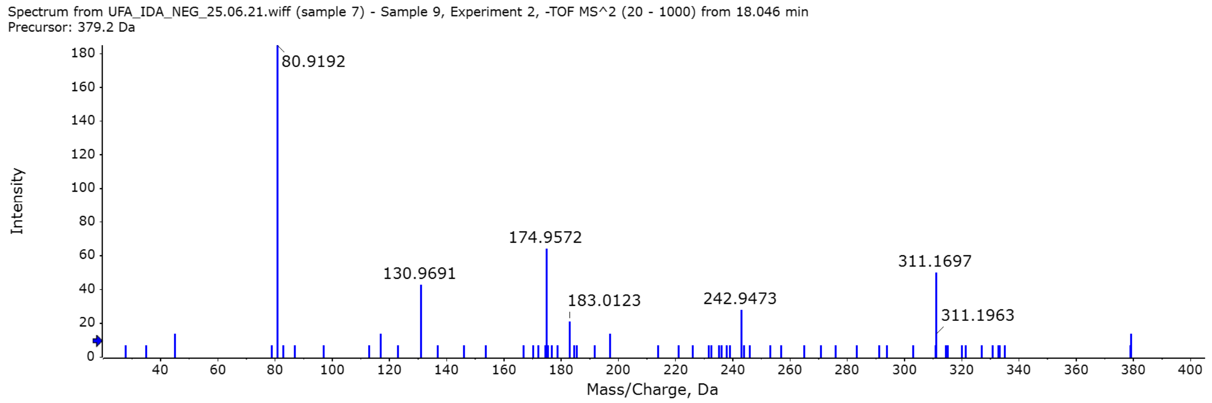

Figure S16. Tandem mass spectrum of the Product 8

Table S7. Found elemental composition of the Product 8

Hit	Formula	m/z	RDB	ppm	MS Rank	MSMS ppm	MSMS Rank	Found
1	$C_{18}H_{35}BrO_3$	377.1697	1.0	-0.5	1	-	1	NA/NA

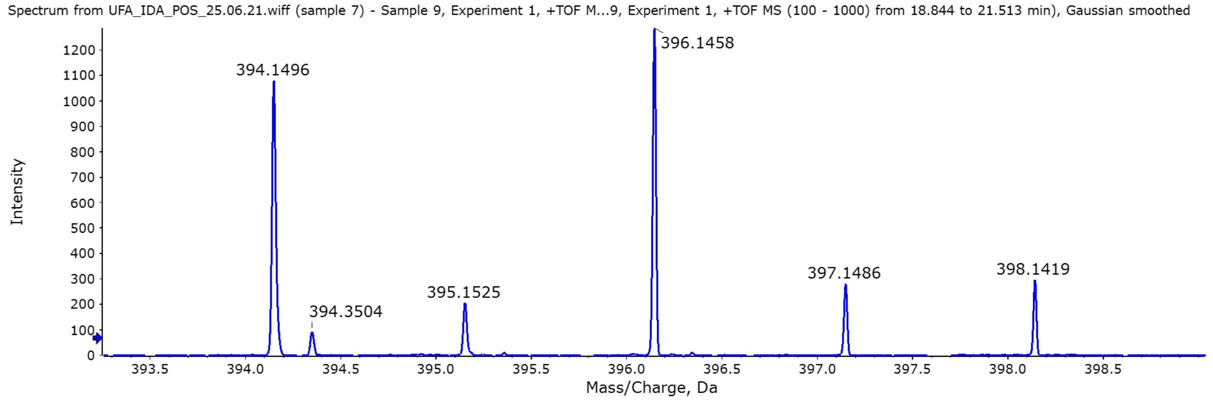

Figure S17. Mass spectrum of the Product 9

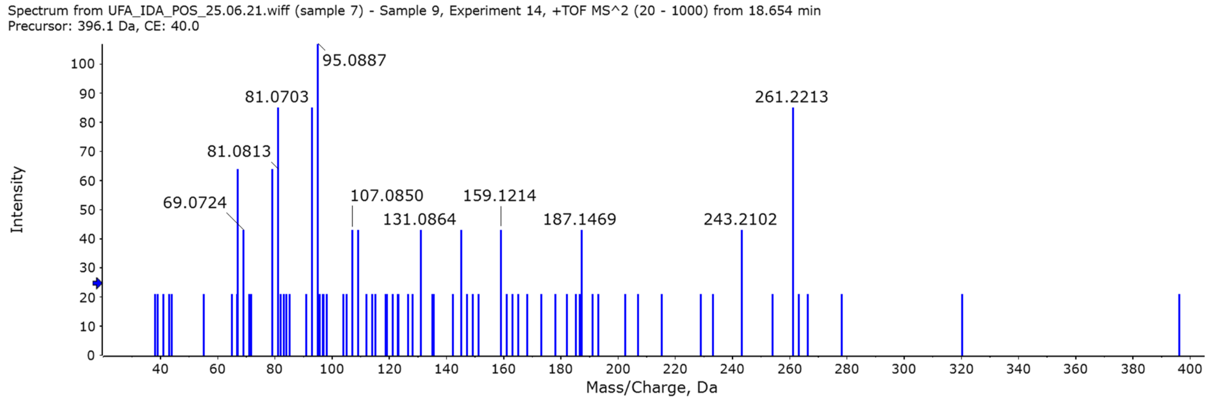

Figure S18. Tandem mass spectrum of the Product 9

Table S8. Found elemental composition of the Product 9

Hit	Formula	m/z	RDB	ppm	MS Rank	MSMS ppm	MSMS Rank	Found
1	$C_{18}H_{33}BrClNO$	394.1507	2.0	-2.7	1	0.9 (2)	1	NA/NA

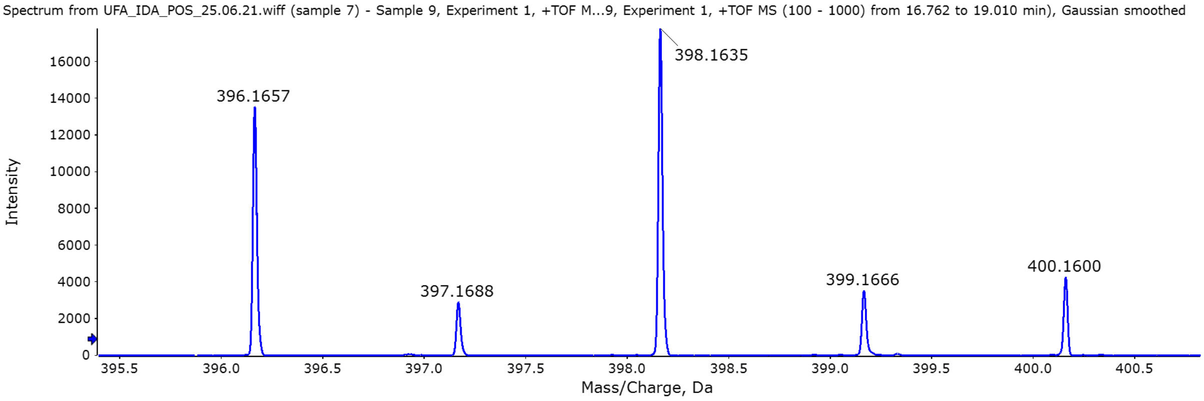

Figure S19. Mass spectrum of the Product 10

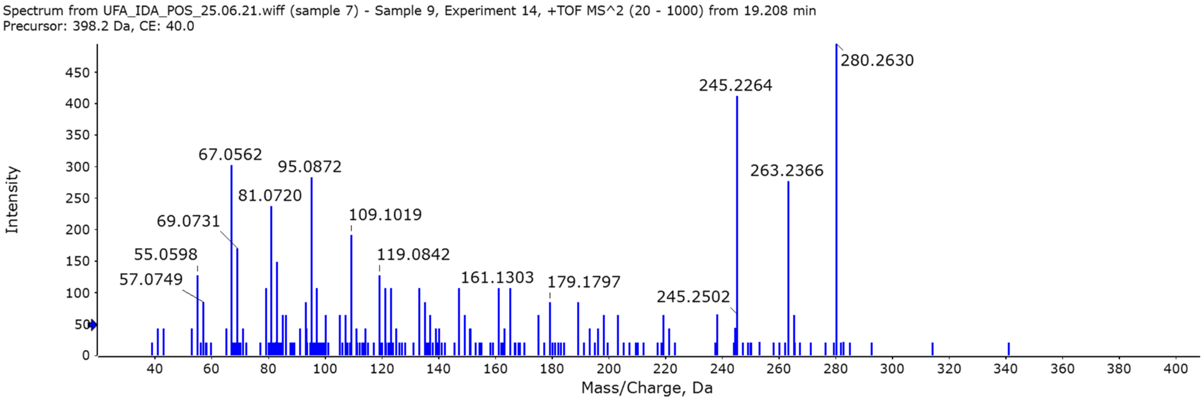

Figure S20. Tandem mass spectrum of the Product 10

Table S9. Found elemental composition of the Product 10

Hit	Formula	m/z	RDB	ppm	MS Rank	MSMS ppm	MSMS Rank	Found
1	$C_{18}H_{35}BrClNO$	396.1663	1.0	-1.6	1	1.8 (10)	1	NA/NA
